# Modified ACDF Technique for the Treatment of Centrum Focal Ossification of the Posterior Longitudinal Ligament: A Case Report

**DOI:** 10.1111/os.13711

**Published:** 2023-03-29

**Authors:** Jing‐lai Xue, Huo‐huo Xue, Wei‐liang Cui, Jing Xiao, Zhong Liao

**Affiliations:** ^1^ Fuzhou Second Hospital Fuzhou China

**Keywords:** Anterior cervical discectomy and fusion, Cervical vertebra, Ossification of posterior longitudinal ligament

## Abstract

**Background:**

Anterior cervical discectomy fusion (ACDF) is a surgical procedure used to treat cervical spondylosis with anterior spinal cord compression. However, there are limitations to traditional ACDF and posterior indirect decompression when the anterior source lesion is in the center of the cervical vertebra.

**Case Presentation:**

On June 8, 2022, our department treated a patient with cervical spondylotic myelopathy—whose high posterior longitudinal ligament (OPLL) occupied the central position of the vertebral body—with modified ACDF. The preoperative surgical plan was designed based on the relevant imaging data and assay index. Also, the visual analogue scale (VAS), Japanese Orthopaedic Association (JOA) scores, and imaging parameters of neck pain were recorded and compared. Postoperative imaging data showed that cervical curvature was recovered and spinal canal compression was relieved. The VAS score for neck pain decreased from 7 preoperatively to 1.5 at the last follow‐up, while the JOA score increased from 10 preoperatively to 29 at the last follow‐up. The volume of the spinal canal was restored. Simultaneously, the patient's extremity muscle strength improved and muscle tension decreased.

**Conclusions:**

Modified ACDF may be an effective surgical method for resolving spinal cord compression in a specific location when bone mineral density is good. We can effectively avoid iatrogenic nerve injury and symptom recurrence by removing the vertebral body and the lesion directly.

## Graphical abstract

Anterior cervical discectomy fusion (ACDF) is often used to treat cervical spondylosis with compression of the anterior part of the spinal cord, but is unable to treat posterior longitudinal ligament (OPLL) with long segments or special locations. We treated a man in June this year who had a specially positioned cervical vertebra OPLL. In the absence of traditional surgical options, we modified the ACDF technique: the intervertebral disc was removed after the anterior longitudinal ligament was exposed, and a small groove was removed above the C5 vertebral body by using an ultrasound osteotomy. After careful separation of the dura and bone mass, the lesion was removed and the cervical spine was fixed. At the last follow‐up, cervical curvature and spinal canal volume recovered. The VAS score of neck pain decreased from 7 points before operation to 1.5 points at last follow‐up. And the JOA score increased from 10 points before operation to 29 points at last follow‐up.

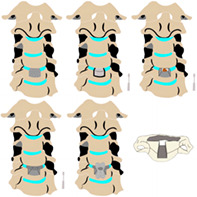

## Introduction

A cause of spinal stenosis (SS) is ossification of the posterior longitudinal ligament (OPLL), which includes intraspinal compression at the corresponding site in iconography. The incidence of OPLL in Asians is 4.8%, which is 1.3% higher than that in Caucasians. Moreover, the cervical vertebra segment is more common in men than in women.^1^ Ossification of the cervical OPLL is mainly characterized by neurological and local symptoms. Although most patients have no obvious symptoms in the early stages, the disease can be diagnosed with CT examination. In later stages of the disease, compression leads to spinal cord degeneration, which appears with central nervous system or urinary system symptoms. During this process of ossification, there are different changes in extremity tendon reflexes, muscle strength, sensory changes, Hoffmann's sign, and Babinski's sign.^2^ Partial cervical OPLL may be associated with local soreness, which can be relieved by traditional treatment in the early stage. Surgical treatment is recommended when serious neurological symptoms occur. There is controversy about choosing a surgical treatment for cervical OPLL. However, satisfactory recovery has been reported with anterior surgery anterior cervical discectomy and fusion (ACDF)/anterior cervical corpectomy and fusion (ACCF), posterior surgery (laminectomy/laminoplasty) and a combination of both surgeries.^3^, ^4^, ^5^, ^6^

Anterior cervical spine surgery can directly remove the compression in front of the spinal cord, which may be due to OPLL ossification, a herniated disk, or proliferative osteophytes. Moreover, it can directly eliminate compression on the spinal cord and effectively restore the spinal canal volume. The improvement rate of neurological function after anterior cervical spine surgery is significantly better than that of laminoplasty, especially for patients with a high proportion of intraspinal ossification (60%) and cervical kyphosis.^7^ However, there are drawbacks. Smaller surgical incisions often pose difficulties with large ossifications and can increase the risk of spinal cord injury.^8^ Regarding centrum focal OPLL with special location, because of the inability of our naked eyes to directly capture the lesion tissue, complete resection of OPLL with poor location cannot be performed in conventional ACDF surgery. Surgical instruments are another reason for the limitation. Currently, even with 45° nucleus pulposus forceps, it is difficult to remove the ossification without damaging the spinal cord. Therefore, special surgical techniques are necessary to expand the spinal canal. The anterior cervical approach to remove the lesion along with part of the vertebral body appears to be a viable option. Therefore, special surgical techniques need to be adopted to expand the spinal canal, and the anterior cervical approach to remove the lesion along with part of the vertebral body appears to be a viable option. The anterior approach, which can lead to nerve injury, cerebrospinal fluid leakage, and insufficient decompression, is still used;^9^ however, some relatively new technical methods have been reported.^10^, ^11^ As a result, surgeons must develop more anatomical knowledge and surgical skills to avoid the occurrence of complications when performing these operations.

This article reports a case of focal OPLL located in the middle of C5. We modified the ACDF procedure and evaluated the short‐term postoperative parameters for reference.

## Methods

### Case Presentation

A 72‐year‐old man presented to the clinic with a complaint of “neck pain accompanied by numbness and weakness of the extremities for more than half a year, which was aggravated for one month.” The patient had a history of hypertension and was taking nifedipine sustained‐release tablets irregularly for antihypertensive therapy. Physical examination revealed that the physiological curvature of the cervical spine became straight, movements in all directions were limited, there was no obvious tenderness, there was percussion pain in each spinous process and paravertebral process, the muscle strength of each limb was grade three, the muscle tension of both lower limbs was slightly increased, the knee tendon and Achilles tendon reflexes were hyperactive, Hoffmann's sign was positive bilaterally, and Chaddock's sign was negative.

**Fig. 1:**
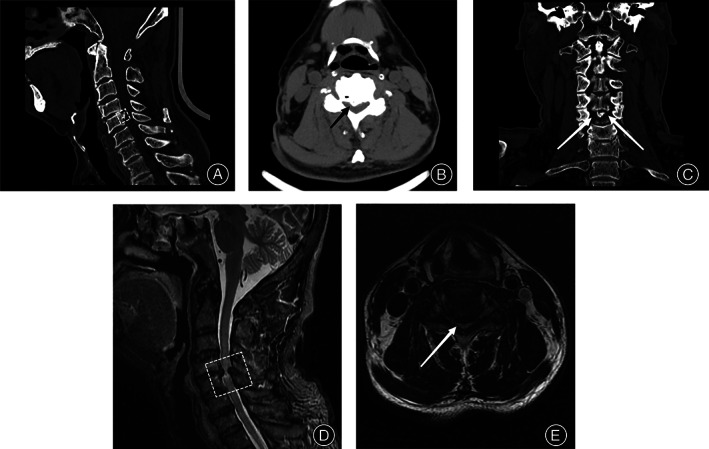
A 72‐year‐old man presented with a complaint of neck pain accompanied by numbness and weakness of the extremities. The muscle strength of each limb was Grade 3, the muscle tension of both lower limbs was slightly increased, and the knee tendon and Achilles tendon reflexes were hyperactive. The following are the preoperative radiology data: (A) The sagittal plane of CT showed that the ossification originated in the middle of the C5 vertebral body, with a width of 4.3 mm and a height of 6.1 mm. (B) The axial plane of CT showed that the ossification was 8.9 mm long and 4.3 mm wide, and the posterior protrusion compressed the spinal canal. (C) The coronal plane of CT showed that the ossification was located in the middle of the vertebral body of C5. (D) Sagittal MRI showed compression of the spinal cord at the C4/5 level, with hyperintensity and suggestive degeneration (the dashed boxes indicate the compressed segments). (E) The anterior and posterior diameter of the spinal canal was 8.1 mm and the OR was 0.6, indicating a high degree of compression

Preoperative radiographs showed multispace degeneration of the cervical spine, with C4/5 being the most serious. The physiological curvature of the cervical spine had become straight. Ossification of the OPLL protruded into the spinal canal at the middle edge of the C5 vertebral body (Fig. 1). C4/5 mild edema was observed in the horizontal spinal cord. Spinal bone mineral density was 1.172 g/cm^2^. The bone mineral density (BMD) T score was 0.7. OPLL of the cervical spine was diagnosed. Cervical spondylotic myelopathy, cervical internal disc herniation (C3/4, C4/5, C5/6, and C6/7), and hypertension were also diagnosed. Preoperative multidisciplinary consultation excluded intolerance to general anesthesia. The surgeon actively improved the preoperative preparation, informed the patient's family, and signed informed consent was obtained.

### Clinical Evaluation

Neck pain visual analogue scale (VAS) scores, Japanese Orthopaedic Association (JOA) scores, and a specialist physical examination were obtained before the surgery. They were reevaluated at 3 days and 45 days after surgery. The results were compared before and after the surgery. The postoperative recovery was comprehensively evaluated.

### Radiologic Evaluation

X‐Rays, CT, and magnetic resonance imaging (MRI) were performed before the operation, and CT evaluation was reviewed after the operation. The following parameters were evaluated: (i) cervical lordosis curvature angle (cervical lordosis, CL): two straight lines were drawn parallel to C2 in the lower middle plate and C7 lower end plate, and a line was drawn perpendicular to the two lines. The angle between them was considered to be the cervical curvature; (ii) recovering degree of the spinal canal space (occupation ratio, OR): the occupying diameter/anterior and posterior diameter of the spinal canal in the axial image of the widest layer of osteophyte on MRI level were used; and (3) CT assessment of the size and length of ossification; MRI was used to evaluate the degree of spinal canal compression and spinal degeneration.

### Preoperative Mentality

The key to this case was early removal of the ossification lesion to eliminate spinal cord compression. The general shape of the ossification was constructed using three‐dimensional CT before surgery. Part of the C5 centrum and ossification were resected with corresponding sizes during the operation, and most of the bony structure was preserved with internal fixation and fusion space. Postoperative attention was paid to the drainage volume of the operative cavity.

### Surgical Procedures

After successful general anesthesia, a transverse incision was made along the C4/5 level of the intervertebral space and into the subcutaneous tissue. The retractor pulls neck muscles, protecting vital blood vessels and exposing the leading edges of C4 and C5.

The posterior longitudinal ligament was opened after fully exposing the centrum and removing the annulus fibrosus and nucleus pulposus of the C4/5 disc. At this point, we could not identify the free bone lesion visually. Therefore, we used an ultrasonic bone knife to remove the central part of the C5 centrum (anteroposterior: 9.4 mm in length and 7.8 mm in width). The bone block groove and most of the OPLL were removed. The remaining ossification was carefully separated from the dural sac. After further clearance of the C4/5 intervertebral space, a cage (filled with cancellous bone and bone meal) of an appropriate size was made and placed in the human intervertebral space. It should be noted that we left a 9.4‐mm long, 20‐mm wide, and 7.8‐mm high cavity without bone graft filling.

**Fig. 2:**
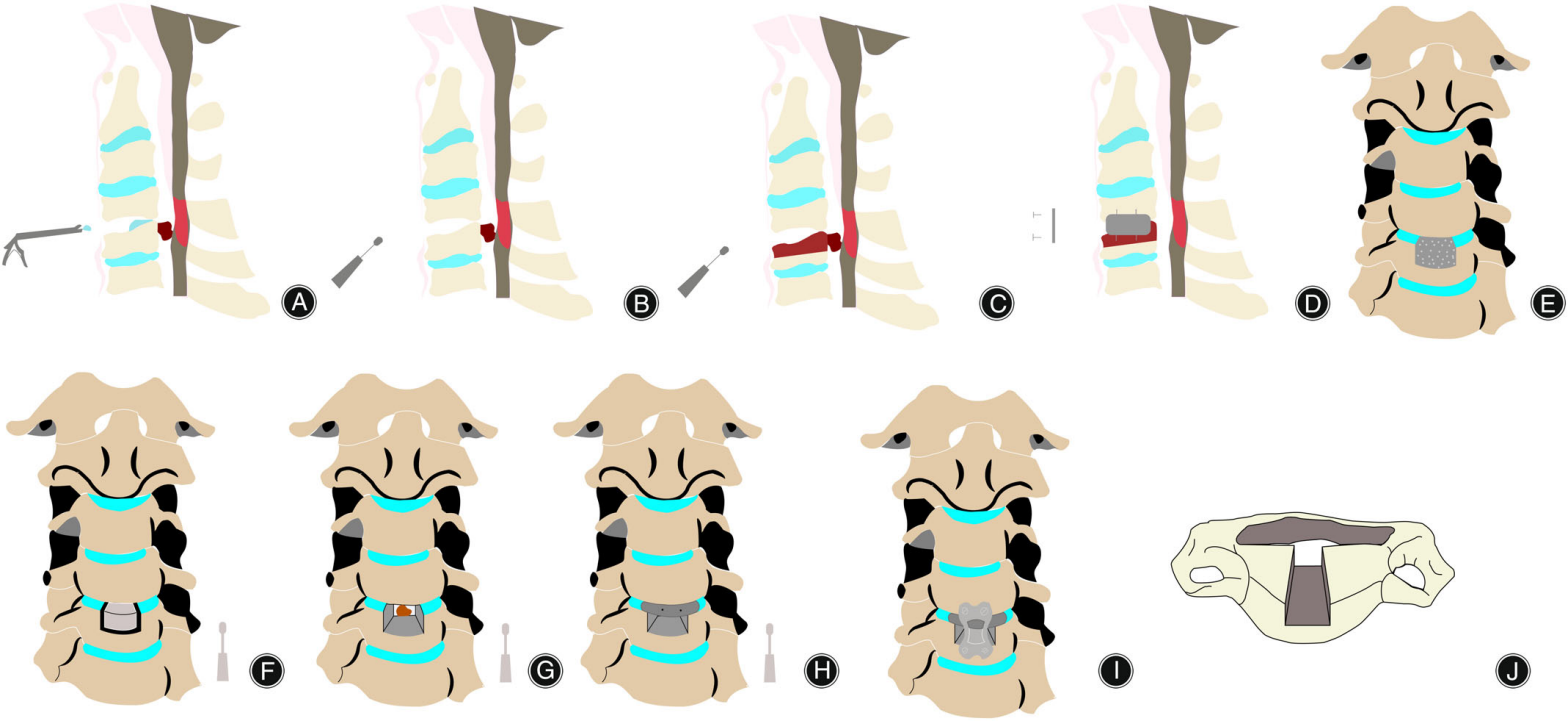
Technical description of the modified ACDF. (A) The posterior longitudinal ligament was opened after the C4/5‐disc tissue was removed with the nucleus forceps. The upper and lower endplates were scraped off with a curette. (B) The upper part of the C5 vertebral body and posterior longitudinal ligament were removed with an ultrasonic bone knife to expose the ossified mass. The ossification was carefully separated from the spinal cord. (C) At this point, we were careful to not cause nerve damage from the surgical instruments, and we removed the ossification. (D) A suitable cage was placed to fuse C4 and C5. A four‐hole titanium plate fixed the position of the vertebral body. (E–I) Coronal fluoroscopy of the surgical procedure. (J) Postoperative residual rectangular cavity, 9.4‐mm long, 20‐mm wide, and 7.8‐mm high

Fluoroscopy was performed again to ensure that there were no other foreign bodies in the spinal canal, and a titanium plate of appropriate size was placed for fixation. The incision was rinsed with saline. After thorough hemostasis, the incision was closed layer by layer, and a pan drainage tube was placed (Fig. 2).

**Fig. 3:**
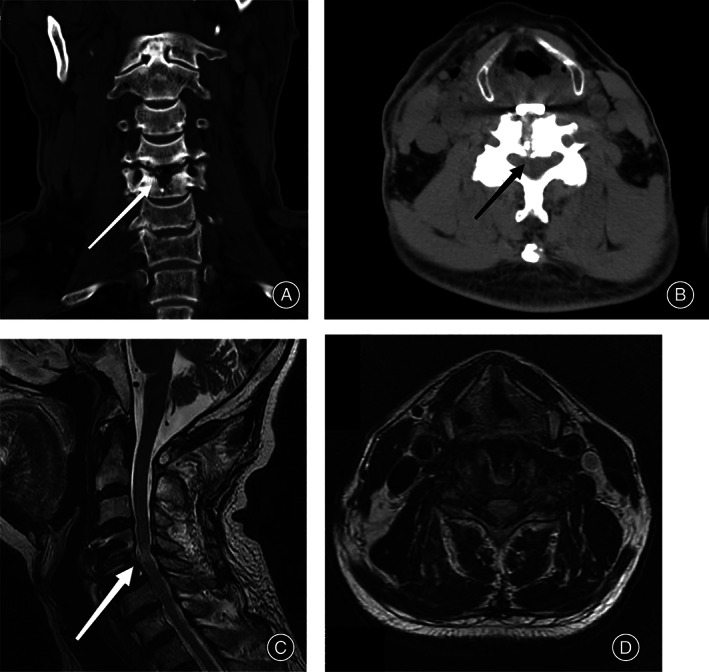
The numbness in the extremities was relieved. Muscle strength in the extremities recovered to Grade 4, and muscle tension improved. The cervical VAS score at 3 days after surgery was 3. The JOA score was 14. The CL angle was 34.99°. The above aspects were better than the preoperative ones. The neck VAS score at 45 days after surgery was 1.9, and the JOA score was 29. The following are the postoperative radiology data: (A) Coronal CT scan 3 days after the surgery showed that there was a bone groove 9.4 mm wide and 7.8 mm long on the C4/5 centrum. (B) In axial CT, the screw fixation was good, the ossification of OPLL was removed, and the OR was 0. (C) At 45 days after the operation, the compression of the spinal cord was relieved according to a sagittal MRI. There was a hyperintense area in the C4/5 level spinal cord. (D) The volume of the spinal canal was recovered, the anteroposterior diameter was 8.1 mm, and the OR was 0. All parameters were significantly better than the preoperative ones

**Fig. 4:**
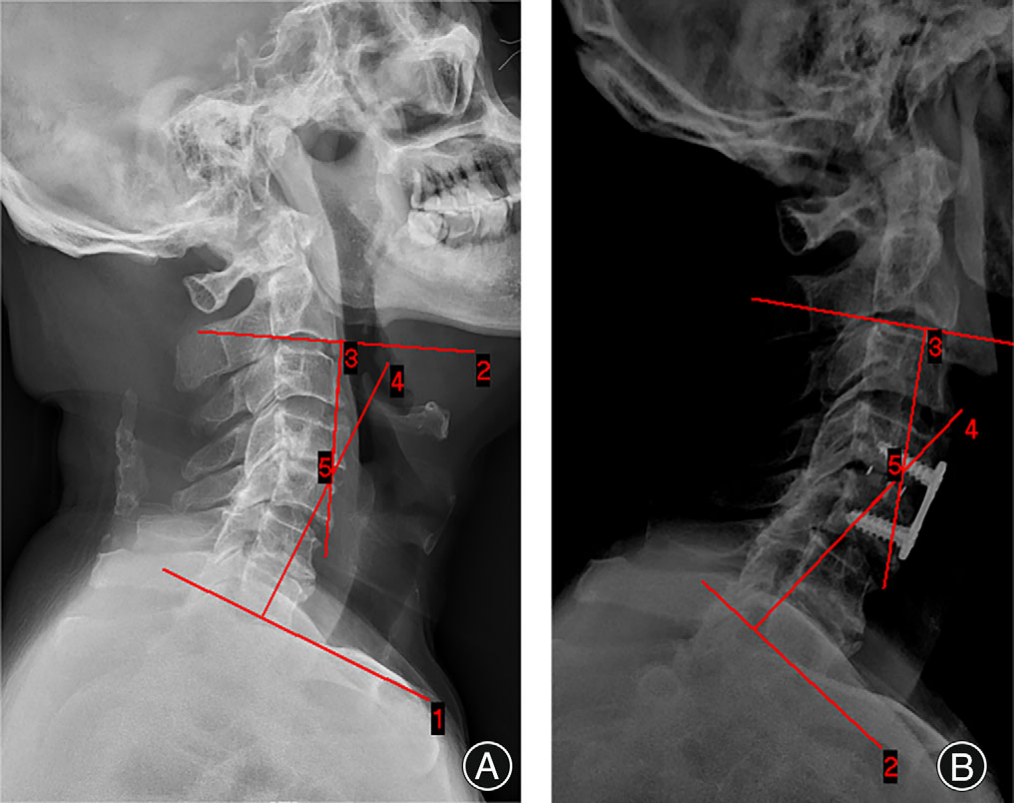
(A) The patient's preoperative CL angel was 22.98°, and the cervical curvature became straight. (B) The patient's postoperative CL angle was 34.99°, and the cervical spine returned to the normal physiological curvature

Dexamethasone and mannitol were used to eliminate nerve edema and prevent inflammation after the operation. Compared with before the operation, the symptoms of numbness in the extremities were significantly improved. The muscle strength of the extremities recovered to Grade 4 after the operation, and muscle tension was improved. Knee and tendon reflexes were observed. Hoffman's sign became negative. Intraoperative blood loss was 24 mL, and hemostasis was performed using gelatin sponge combined with bone wax. The postoperative drainage volume was approximately 32 ml. The negative pressure drainage vessel was removed 2 days after the operation, and the neck was fixed with a neck brace. The preoperative VAS score for neck pain was 7, and the JOA score was 10. The CL angle was 22.98°, and the OR was 0.6. The postoperative VAS score for neck pain was 3, and the JOA score was 14. The CL angle was 34.99°, and the OR was 0. A review of postoperative imaging showed recovery of the spinal canal space (Fig. 3). The physiological curvature of the cervical spine was improved after the operation compared with that before the operation (Fig. 4). All quantitative scores were better than those before the operation and were similar to those of previous patients with ACDF.^12^, ^13^ There were no related complications in this case.

## Discussion

### Review of the Literature on Focal Ossification of the Posterior Longitudinal Ligament

The pathogenesis of cervical OPLL is complex and is generally considered to be affected by a variety of physical and chemical factors. In recent years, Chinese research teams have found that ligament cells with abnormal OPLL highly express miR‐320 e and affect the expression of TAK1 in ligament cells and mesenchymal stem cells by secreting disease specific sEV, which makes ligament cells more likely to generate an osteogenic differentiation and inhibit osteoclast differentiation of monocytes.^14^ Most of the reports on OPLL are concentrated in Asia, and the ethnic differences are closely related to the incidence of OPLL.^1^ Among them, the more obvious symptoms of cervical posterior longitudinal ligament ossification have attracted the attention of many people. The Japanese researchers divided OPLL into four types: focal, segmental, continuous, and mixed.^15^ Focal OPLL is characterized as a single discontinuous ossified mass, generally situated adjacent to the vertebral space. Isolated ossified masses in the posterior margin of the vertebral body are uncommon and are usually classified as segmental OPLL. Anterior surgery (ACDF and ACCF) is a traditional surgical procedure for the radical treatment of focal OPLL or vertebral posterior bone mass (Table 1). These methods directly eliminate premyelopathy and decompress the spinal canal through an anterior cervical approach, and similar treatment experiences have been shared globally.^10^, ^11^, ^16^, ^17^, ^18^, ^19^, ^20^, ^21^, ^22^, ^23^

**TABLE 1:** Surgical methods and therapeutic effects of OPLL reported in the literature

Authors	Year	Group	OPLL Type	Surgical treatment	Result
Chen *et al*.^17^	2009	With/without dural ossification (DO)	Focal	ACCF	Patients with DO had extreme postoperative clinical scores and were more likely to have cerebrospinal fluid leakage. In response to such cases, the drainage tube should be removed as soon as possible and the wound should be pressed.
Chen *et al*.^18^	2009	With/without dural ossification (DO)	Focal	ACCF	Cervical lordosis, flattening rate, and JOA score were enhanced after surgery, while the proportion of DO and vertebral canal occupied were lower after single‐level.
Dalbayrak *et al*.^21^	2010	“Skip” corpectomy/Laminoplasty	Segmental	ACCF was conducted on adjacent segments of the target vertebral body.	Improvement in cervical lordosis in 26 patients was 14.36° ± 7°, and bone fusion was performed in all patients.
Lei *et al*.^20^	2016	Enlarged ACDF	Focal	Posterior margin of the upper and lower vertebrae of the OPLL‐ossified mass segment was removed, and the OPLL‐ossified mass was entirely removed.	Mean operation time was 108.1 ± 21.6 min, and the mean blood loss was 173.3 ± 57.1 ml. JOA score at the 3‐month follow‐up was 13.5, and the improvement rate was 64.3% ± 15.1%.
Hou *et al*.^22^	2017	ACDF/Laminoplasty	Segmental/Focal	ACDF/Laminoplasty	Compared with laminoplasty, ACDF had better JOA and VAS scores and fewer postoperative complications; the physiological curvature of the cervical spine recovered better.
Sun *et al*.^10^	2018	Anterior controllable antidisplacement and fusion (ACAF)	Segmental	Posterior longitudinal ligament complex (VOC) of the vertebral body was segmented intraoperatively, and anterior plates and screws were installed. After bilateral osteotomy of the vertebral body, VOC was lifted using the Bridge Crane technique.	Occupation ratio and cervical lordosis of all patients appeared to be normal after surgery, and the clinical scores were enhanced to varying degrees than those before surgery.
Xu *et al*.^19^	2019	ACCF/laminoplasty	Segmental	ACCF/laminoplasty	ACCF's final follow‐up JOA score advanced with its final follow‐up recovery rate over laminoplasty.
Zekaj *et al*.^16^	2020	Vertebral endplate osteotomy technique	Segmental	Posterior upper and posterior lower segments of the involved vertebra were excised and fixed with bidirectional screws and plates.	Postoperative neurological symptoms were alleviated in all patients, and 15 months later, bone fusion was suggested without CSF leakage.
Lee *et al*.^11^	2020	Vertebral body sliding osteotomy (VBSO)	Segmental	Posterior longitudinal ligaments of both ends of the vertebral bodies were totally cleared, and the intervertebral space of the implicated segments was fused. Vertebrae‐OPLL complex was manually lifted after osteotomy at both ends of the vertebral body.	JOA score, postoperative cervical sagittal angle, and sagittal cervical lordosis angle were all improved at the last follow‐up. All patients achieved bone fusion 2 years after surgery with no complications.
Noh *et al*.^23^	2020	ACDF/Laminoplasty	Focal	ACDF/Laminoplasty	Compared with laminoplasty, ACDF had a higher positive clinical score; achieved better postoperative recovery of C2–C7 Cobb angles and segmental angle; and resulted in a greater reduction in the spinal cord occupation ratio.

Among them, ACDF is suggested only for treating posterior longitudinal ligament injuries near the intervertebral space. When the lesion is in a blind area inaccessible by medical instruments, conventional ACDF is inadequate. ACCF is more severe and involves the resection of a multilevel vertebra, which has a direct impact on the resection of OPLL at the posterior edge of the vertebra. However, despite dural ossification, the treatment of the supradural bone slice still needs to be concentrated on, and this technology has no evident advantages in the length of the postoperative hospital stay, bleeding, or drainage. Therefore, isolated posterior margin OPLL requires a less invasive surgical solution.

### Surgical Technique Selection and Modified Technique

The minimally invasive concept of modern spinal surgery emphasizes minimizing the invasive and destructive nature of the operation. A surgically appropriate approach should be more targeted.^24^ Therefore, the ACCF with more trauma or indirect decompression must be handled with a posterior approach. Moreover, a posterior indirect decompression injury is more extensive. Additionally, the decompression effect of high spinal canal occupancy or cervical kyphosis was not obvious in this case. As a result, these findings were inconsistent with our minimally invasive philosophy.

In this case, some OPLL isolated compression symptoms were severe and the efficacy of posterior decompression was uncertain. Therefore, both anterior ACDF and ACCF had their own limitations. Zekaj *et al*. have performed partial vertebral excisions in the horizontal direction of ossification in a case of multisegment OPLL at the C3/4 and C4/5 level and fixed it with two‐way screws and an anterior plate.^16^ This provided the idea for our surgical design. In this case, we decided to perform a modified ACDF procedure. The preoperatively designed protocol was used after curettage of the C4/5 disc. Part of the central C5 upper endplate and part of the vertebral body (a rectangular slot with a length of 9.4 mm and a height of 7.8 mm, slightly larger than the lesion) were excised using an ultrasound osteotomy. Finally, the ossified lesion was excised, and the bony cavity was retained.

### Postoperation Considerations

There were still some issues to consider. As the bone growth relied on osteoconduction and osteoinduction,^25^ new bone tissues and blood vessels would grow toward the bone graft area in the cage, and bone conduction would be formed at the contact point between the cage and the endplate. At the same time, these situations would promote bone induction: postoperative local biomechanical changes of cervical spine, increased stress on both sides of the bone graft, and mechanics. According to Wang *et al*.,^26^, ^27^ several bone marrow cells in the bone could produce osteoinduction and promote osteogenic differentiation when stimulated by appropriate mechanical stress. This may cause faster bone growth in both vertebral bodies. However, the increased stress on both vertebral bodies may cause a risk of vertebral collapse.

Cage subsidence is another issue to consider. The cage is embedded in the vertebral body after vertebral fusion for various reasons and can result in the direct loss of intervertebral height. It is now widely accepted that bone density and age are the main risk factors for spinal fusion cage subsidence, and excessive body mass index is an important cause of increased cage surface pressure.^28^, ^29^ Therefore, it is necessary to adequately assess bone quality before surgery. The diseased vertebra reported here was located at C5. The cervical cage has a lower weight‐bearing pressure unit compared with the lumbar cage, and the higher bone density allows the residual endplate to support the cage. However, according to Pinter *et al*.'s report,^30^ age can be a risk correlation factor in predicting moderate or even severe cage subsidence. The risks of both vertebral collapse and cage subsidence are highly correlated with BMD, with age having a greater correlation with BMD. Briefly, as the follow‐up duration increases, aging and changes in bone mass increase the risk faced by patients. Although we have not been able to find evidence of subsidence on this patient's CT films at this time, we still need to follow his radiological manifestation for a long period after the surgery, instruct him to undergo regular bone density examinations, and contact him promptly for antiosteoporosis treatment in case of poor bone mass manifestation.

### Intraoperative Precautions and Postoperative Conditions

A preoperative three‐dimensional CT scan was performed to determine the location and size of the bone lesion (length 8.9 mm, width 4.3 mm, and height 6.1 mm). The surgical plan was designed by reconstructing the bone's shape and anatomical relationship. The preoperative MRI revealed the soft tissue conditions around the bone lesion, and damage to the spinal cord was avoided as much as possible during intraoperative decompression. Before the vertebra was slotted, the notch size was made slightly larger than the lesion size. At the same time, the two ends of the groove could not exceed the fused space on both sides of the cage. We ensured that the medical equipment could remove the bone lesion while reserving the fusion space. An ultrasonic bone knife was used to remove the bone during slotting, and the operation process carefully avoided straying into the vertebral canal and causing injury. At the time of reaching the posterior longitudinal ligament, the bone lesion and dural sac were carefully separated with a nerve stripper and removed with nucleus pulposus forceps. A cage of appropriate size was used for fusion. The two sides of the cage were placed on the reserved bone space on both sides of the vertebral body. A pre‐bent titanium plate was placed in front of the centrum to maintain the physiological curvature. The results showed that the postoperative drainage volume and postoperative VAS and JOA scores of neck pain in this case were similar to those of previous ACDF procedures. The patient's symptoms were better than those before the operation, and the curative effect was satisfactory.

### Surgical Complications

In terms of surgical complications, postoperative hematoma, iatrogenic injury of nerve tissue, and dural tears are more common in anterior surgery. The incidence is 5.6% of postoperative hematomas in anterior surgery.^31^ This situation mainly stems from anterior soft tissue and centrum small blood vessel injury; however, it can be eliminated with adequate drainage and absorption. Intraoperative C5 nerve root injury is an aspect of iatrogenic nerve injury, with an incidence of 1.6%–12%.^32^ Centrum grooving wounds >15 mm may lead to a higher risk of C5 nerve injury. We think that this may be due to disruption of the venous network in the centrum affecting the effective perfusion of nerves. The incidence of cerebrospinal fluid leakage is 0.5%.^31^ The reason may be the adhesion of the spinal dura mater with ossification or even ossification of the spinal dura mater. In order to fully decompress, it is necessary to remove the adhesion or ossified dura during dissection, which easily produces cerebrospinal fluid leakage. Careful identification of the double‐contour sign on CT before the operation is helpful to judge dural ossification and adopt preventive measures. It can also help manage intraoperative complications carefully. At present, there are also reports on complications after anterior cervical vertebral surgery, such as swallowing dysfunction,^33^, ^34^ hypoglossal nerve palsy, implant infection, and adjacent vertebral lesions. Fortunately, no similar postoperative complications occurred in this case.

### Limitations

This study has certain limitations. At present, there is only one completed case, which is not representative. The biggest controversy in this study is related to the surgery itself. The likelihood of the vertebral body collapsing and cage subsidence are issues that we shall continue to focus on in the future. We need to conduct multicenter randomized controlled trials with a longer follow‐up period and more surgical cases to comprehensively evaluate the feasibility of the new technology.

### Conclusion

The present study showed that the special place of focal OPLL at the posterior margin of the cervical vertebral can be resolved with a modified ACDF procedure. This technique can directly remove the anterior source of spinal cord compression, thereby reducing the destruction of skeletal structures and inadequate decompression. The postoperative functional scores and drainage volume were comparable to those of previous ACDF cases. Therefore, ACDF can be used as an appropriate reference when dealing with cases similar to single focal OPLL compression.

## Author Contributions

ZL designed the technique. HHX and XJL contributed equally to this manuscript. HHX designed the illustrations. WLC, JX collected relevant imaging data and translated the manuscript. All authors read and approved the final manuscript.
